# Allergic rhinitis is associated with thromboembolic disease in pregnancy

**DOI:** 10.1038/s41598-022-11398-z

**Published:** 2022-05-04

**Authors:** Chia-Ta Wu, Chien-Han Tsao, Kuan-Ting Chen, Yu-Tzu Lee, Min-Sho Ku

**Affiliations:** 1 Department of Emergency Medicine, Changhua Christian Hospital, Changhua, Taiwan; 2 School of Medicine, Chung Shan Medical University, Taichung, Taiwan; 3 Institute of Medicine, Chung Shan Medical University, Taichung, Taiwan; 4 Department of OBS/GYN, Jin-Sin Women and Children’s Hospital, Tainan, Taiwan; 5 Division of Allergy, Asthma and Rheumatology, Department of Pediatrics, Chung Shan Medical University Hospital, Taichung, Taiwan

**Keywords:** Diseases, Risk factors

## Abstract

Finding the risk factors for thromboembolic (TE) disease and preventing its development in pregnant women is important. Allergic rhinitis (AR) is a common chronic disease. We aim to find if AR is a risk factor. From 2004 to 2011, 55,057 pregnant women were recruited from a Taiwan database. They were grouped into AR and non-AR groups. The rate of TE and venous complications during pregnancy and 60 days after childbirth were compared between non-AR and the AR group. Those with AR diagnosed both before and after childbirth, meaning AR was not changed during pregnancy, the rates of TE (OR 2.64) and venous complications (OR 1.35) were higher compared to non-AR subjects. In those who underwent cesarean delivery, the rate was also higher in group 3 (OR 4.14). Those with AR before childbirth, without after, meaning AR was well controlled during pregnancy, the rate of TE was not higher than that of the non-AR subjects. Pregnant women with AR have an increased rate of TE. An increased rate of venous complications in these subjects might explain the increase in TE. If AR is well controlled during pregnancy, the rate of TE does not appear to increase.

## Introduction

Pregnant women have an increased risk of developing thromboembolism (TE) disease compared with non-pregnant women of the same age^1,2^. TE, including pulmonary embolism (PE), was one of the main causes of maternal death during pregnancy in developed countries^2,3^. However, the symptoms and signs of TE and PE are non-specific, and the diagnosis may be delayed or missed. Earlier diagnoses of TE or PE could reduce the morbidity and mortality^4^. Therefore, in pregnant women, finding the risk factors for VTE (venous thromboembolism) may remind physicians to pay more attention to these patients and then find TE earlier.

Previous studies indicated that allergic disease is associated with pro-thrombotic alterations. Activation of the coagulation system during allergic response^5^, enhanced platelet activation^6^ and formation of dense fibrin clots^7^ were reported to be the underlying mechanisms. Immunoglobulin E (IgE), a biological marker of allergic disease, was reported to exert some prothrombotic and antifibrinolytic activities^8^.

Twenty to thirty percent of pregnant women are affected by allergies. Allergic symptoms existing before pregnancy may be either attenuated, or equally often promoted through pregnancy^9,10^. Total IgE levels, a predictor of the severity of allergic disease, also change before, during and after pregnancy^11^. As mentioned before, the risk of embolic disease has been correlated with the severity of allergic disease. However, in pregnant women, when the severity of their allergic disease attenuates during pregnancy, does the risk of embolic disease decrease? When the severity of their allergic disease is promoted during pregnancy, does the risk of embolic disease increase? This data has not been studied before.

Allergic rhinitis (AR) is a common allergic disease in adults. An association between AR and TE has been reported^12^. However, the association between AR and TE in pregnant women has not been researched up to now. The aim of this study is to understand if AR improves or worsens during pregnancy and if the rate of TE increases or decreases by comparing the rate of TE between non-AR subjects and subjects with AR.

## Methods

### Database and data collection

**Figure 1:**
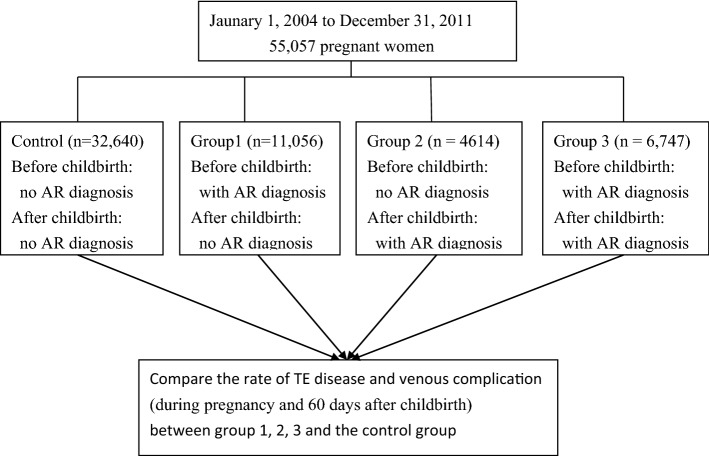
Flow chart.

A representative sample of 1 million participants from the National Health Insurance Research Database was provided by Taiwan’s National Health Research Institutes^13,14^, and this sample served as our data source. Information on basic patient demographics and medical care received, including outpatient, inpatient claims and childbirth was gathered. The study flowchart is presented in Fig. 1.The institutional review board of Chung Shan Medical University Hospital, Taichung, Taiwan, approved this study (ethics approval number: CS2-20161). All experiments were performed in accordance with relevant guidelines and regulations. The data taken from the National Health Insurance Research Database was anonymised before its use in the study. The International Classification of Diseases, 9th Revision, Clinical Modification (ICD-9-CM) was used to identify diagnoses of diseases in the claims data. The ICD-9-CM code used in our study is presented in Table 1.

**Table 1:** ICD-9-CM codes used in the study.

Disease	ICD-9-CM code
**Allergic disease**	
AR and AC	477, 372.05, 372.14
AS	493
**Thromboembolism**	
TE of intracranial venous sinuses	325
Pulmonary embolism and infarction	415.1
TE of arteries, arterioles and capillaries	444, 445
Venous TE	451, 452, 453
Venous TE in pregnancy and puerperium	671.2 ~ 671.5
Obstetrical pulmonary embolism	673.2, 673.8
**Medical comorbidities**	
Type I DM with nephropathy	250.41, 250.43
Chronic heart disease	393–398, 424–429
Systemic lupus erythematosus	695.4, 710.0
Inflammatory bowel disease	555, 556
Obesity	278
Thrombophilia	286.53, 289.81, 289.82
Nephrotic syndrome	581
Tuberculosis	010 ~ 018
Inflammatory polyarthropathy	716.59
**Pregnancy characteristics**	
Multiple gestation	651
Preeclampsia	642.4 ~ 642.7
Pregnancy-related hypertension	642.0 ~ 642.3, 642.9
**Delivery complications**	
Early or threatened labor	644
Postpartum hemorrhage	666, 667
Prolonged labor	662
Infection (7 days before ~ 60 days after childbirth)	590.1, 590.8, 590.9, 595.0 ~ 595.4, 595.9, 647, 658.4, 659.2, 659.3, 670.0, 672, 675, 5990, 486, 998.59
**Venous complications in pregnancy and the puerperium**	671

### Recruitment and grouping of subjects

From 2004 to 2011, 55,057 pregnant women were selected for the study. To prevent the complex conditions of patients that would influence the results of the study, those with the diagnosis of malignancy, major illnesses such as chronic psychosis, paralysis or severe trauma before childbirth were excluded from the study. The claim data of these subjects, 3 years before and 2 years after childbirth were gathered for the study. Those with and without the diagnosis of AR during the 5 years were grouped into the AR group and non-AR group. The AR subjects were further divided into three groups. Group 1: with AR before; without AR after childbirth (well controlled AR after childbirth). Group 2: without AR before; with AR after childbirth (poor control of AR or new onset of AR). Group 3: with AR before; with AR after childbirth. The criterion for “with AR before childbirth” was: at least one diagnosis of AR 3 years before childbirth. The criterion for “with AR after childbirth” was: at least one diagnosis of AR 2 years after childbirth. TE selected in our study is presented in Table 1.

### Rate of TE

Those with TE diagnosis were counted as “with TE”. The criteria of “with TE” were: had one or more TE diagnosis during pregnancy and 60 days after childbirth. The rates of TE were compared between AR and non-AR subjects. The rates of TE in groups 1, 2 and 3 were also compared to the non-AR subjects. In those with cesarean delivery, the rates of TE were also compared between groups.

### Rate of venous complications

Those with “venous complications in pregnancy and puerperium” were counted as “with venous complications”. The criteria of “with venous complications” were: had one or more venous complication diagnosis during pregnancy and 60 days after childbirth. The rates of venous complications in groups 1, 2 and 3 were compared to those of the non-AR subjects.

### Determining the confounding factors

According to the guidelines of the Royal College of Obstetricians and Gynecologists (RCOG)^15^, risk factors for TE during pregnancy and puerperium were viewed as the confounding factors. These confounding factors include birth type (cesarean versus vaginal delivery), mother’s age (less than versus equal or more than 35 years old). Other factors including medical comorbidities, pregnancy characteristics and delivery complications are presented in Table 1. Socioeconomic status, urbanization that might influence characteristics of pregnancy and delivery were also considered as the confounding factors. Socioeconomic status was defined according to occupation, which was grouped as middle to high- socioeconomic status (teacher or public official, company employee) and low- socioeconomic status (other, peasants or fisherman, low income or no fixed job). Based on Liu’s report^16^, urbanization levels were grouped into seven levels: levels 1–2 for urban residents, and levels 3–7 for country residents. Asthma (AS) was considered to be a risk factor of TE^17^, and was then adjusted. The criterion of AS was: at least 2 AS diagnoses during the 5 years.

### Statistical analysis

All statistical analyses were performed using SAS software version 9.1 (SAS Institute Inc., Carey, NC), and PASW Statistics 18 (IBM, Armonk, NY, USA). The chi-square test was used to compare the rate of TE between groups. Multivariate logistic regression analyses were used for adjusting for the confounding factors. Two-sided *p* values of < 0.05 were defined as significant.

## Results

### Study flowchart and demographic data

The study flowchart is presented in Fig. 1. The demographic data of AR and non-AR groups is presented in Table 2. A total of 55,057 women were recruited, 22,417 (40.72%) were diagnosed with AR. The frequency of country residence and low socioeconomic status were higher in the non-AR subjects. The rate of cesarean delivery, medical comorbidities, pregnancy characteristics, delivery complications were higher in AR groups. The co-existence of AS was higher in AR subjects (6.30%), compared to the non-AR subjects (1.93%).

**Table 2:** Demographic data.

	AR (*n* = 22,417)	Non-AR (*n* = 32,640)	*p*-value
Urban	17,276 (77.07%)	24,837 (76.09%)	0.008
Country	5141(22.93%)	7803 (23.91%)	
**Socioeconomic status**			0.000
Middle to high	18,786 (83.80%)	26,657 (81.67%)	
Low	3631 (16.20%)	5983 (18.33%)	
Cesarean delivery	8287 (36.97%)	11,719 (35.90%)	0.011
Vaginal delivery	14,130 (63.03%)	20,921 (64.10%)	
**Medical comorbidities**			0.000
Yes	797 (3.56%)	742 (2.27%)	
No	21,620 (96.44%)	31,898 (97.73%)	
**Pregnancy characteristics**			0.046
Yes	890 (3.97%)	1188 (3.64%)	
No	21,527 (96.03%)	31,452 (96.36%)	
**Delivery complications**			0.000
Yes	6917 (30.86%)	9144 (28.01%)	
No	15,500 (69.14%)	23,496 (71.99%)	
Asthma	1412 (6.30%)	630 (1.93%)	0.000
No asthma	21,005 (93.70%)	32,010 (98.07%)	
**TE**			0.264
Yes	22 (0.98‰)	23 (0.70‰)	
**Venous complications**			0.113
Yes	169 (7.54‰)	209 (6.40‰)	

### Rate of TE between AR and non-AR pregnant women

The rate of TE in the AR group was 0.98 per 1000 subjects (0.98‰), and the rate in the non-AR group was 0.70 per 1000 subjects (0.70‰). The *p* value was 0.264, OR 1.39; and 95% CI (confidence interval) 0.78 ~ 2.50. After adjusting for the confounding factors, the p-value was 0.521. The rate of venous complications in the AR group was 7.54‰, and the rate in the non-AR group was 6.40‰. The *p* value was 0.113 (OR 1.39; 95% CI 0.96 ~ 1.45. After adjusting for the confounding factors, the *p* value was 0.104. There was no difference.

### Rate of TE in different AR groups

The results of TE in different AR groups are presented in Table 3. In those with AR both before and after childbirth (group 3), the rate of TE was 1.78‰. In those without AR diagnosis (non-AR group), the rate of TE was 0.67‰. Group 3 had a higher TE rate than the non-AR group (*p* = 0.005, OR 2.64, 95% CI 1.31 ~ 5.34). After adjusting for the confounding factors, the *p* value = 0.027. There was no difference between group 1 (with AR before; without AR after childbirth), group 2 (without AR before; with AR after childbirth) and the non-AR group.

**Table 3:** Rate of VTE in different AR conditions.

Group	VTE rate (case/total, ‰)	*p*-value	Odds ratio	95% CI	Adjusted *p*-value
Control	22/32,640 (0.67‰)				
Group1	8/11,056 (0.72‰)	0.863	1.07	0.48 ~ 2.41	0.880
Group2	3/4,614 (0.65‰)	0.953	0.97	0.29 ~ 3.22	0.930
Group3	12/6,747 (1.78‰)	**0.005**	**2.64**	**1.31 ~ 5.34**	**0.027**

Significant values are in bold.

Group 1: with AR before; without AR after childbirth.

Group 2: without AR before; with AR after childbirth.

Group 3: with AR before; with AR after childbirth.

Adjusted *p*-value: adjusted for AS, medical comorbidities, pregnancy characteristics, delivery complications, urbanization, jobs, age and birth type.

### Rate of venous complications in different AR groups

The results of venous complications in different AR groups are presented in Table 4. In group 3, the rate of venous complications was 8.60‰. In the non-AR group, the rate of venous complications was 6.40‰. The venous complication rate was higher in group 3, compared to that in the non-AR group (*p* = 0.046, OR 1.35, 95% CI 1.01 ~ 1.80). After adjusting for the confounding factors, *p* value = 0.037. The rate was similar between group 1, group 2 and the non-AR group.

**Tabel 4:** Rate of venous complications in pregnancy and puerperium in different AR conditions.

Group	Complication rate (case/total, ‰)	*p*-value	Odds ratio	95% CI	Adjusted *p*-value
Control	209/32,640 (6.40‰)				
Group1	75/11,056 (6.78‰)	0.667	1.06	0.81 ~ 1.38	0.628
Group2	36/4,614 (7.80‰)	0.271	1.22	0.86 ~ 1.74	0.279
Group3	58/6,747 (8.60‰)	**0.046**	**1.35**	**1.01 ~ 1.80**	**0.037**

Significant values are in bold.

Group 1: with AR before; without AR after childbirth.

Group 2: without AR before; with AR after childbirth.

Group 3: with AR before; with AR after childbirth.

Adjusted *p*-value: adjust for AS, medical comorbidities, pregnancy characteristics, delivery complications, urbanization, jobs, age and birth type.

### Rate of TE in those who underwent cesarean delivery

For those who underwent cesarean delivery, the rates of TE in different groups are presented in Table 5. In group 3, the rate of TE was 3.88‰. In the non-AR group, the rate was 0.94‰. Group 3 had a higher TE rate than the non-AR group (*p* = 0.000, OR 4.14, 95% CI 1.76 ~ 9.77). After adjusting for the confounding factors, *p* value = 0.006. The rate was similar between group 1, group 2 and the non-AR group.

**Table 5:** Rate of TE in different AR conditions in CS subjects.

Group	VTE (case/total, ‰)	*p*-value	Odds ratio	95% CI	Adjusted *p*-value
Control	11/11,718 (0.94‰)				
Group1	5/4,020 (1.24‰)	0.600	1.33	0.46 ~ 3.82	0.919
Group2	3/1,690 (1.77‰)	0.320	1.89	0.53 ~ 6.79	0.362
Group3	10/2,578 (3.88‰)	**0.000**	**4.14**	**1.76 ~ 9.77**	**0.006**

Significant values are in bold.

Group 1: with AR before; without AR after childbirth.

Group 2: without AR before; with AR after childbirth.

Group 3: with AR before; with AR after childbirth.

Adjusted *p*-value: adjusted for AS, medical comorbidities, pregnancy characteristics, delivery complications, urbanization, jobs and age.

## Discussion

In this population based, retrospective cohort study, we did not find an association between AR and thromoembolic (TE) disease in pregnant women. When we divided the AR subjects into those with AR diagnosis both before and after childbirth (group 3), their rate of TE was higher compared to the non-AR subjects. The increased rate was more prominent in women who underwent cesarean delivery (OR 2.64 in all women and 4.14 in women with cesarean delivery). In these subjects (group 3), the rate of venous complications was also higher. Venous complications including varicose veins were risk factors for TE. From the findings, we speculate that through the increased occurrence of venous complications, women with AR diagnosed both before and after childbirth had a higher rate of TE when they were pregnant.

AS and AR often coexist. AS is associated with a pro-coagulant state^7^. The risk of pulmonary embolism and deep venous thrombosis also increase in AS patients^18^. Therefore, whether the association between AR and TE in pregnancy comes from the cofactor of AS or not, is questionable. In our study, after adjusting for AS, the rate of TE in pregnancy was still higher in those who had AR diagnosed both before and after childbirth (group 3). Therefore, AR itself was the risk factor for TE in pregnancy.

Vaso-active compounds (histamine, tryptase and other cytokines) released from mast cells play an important role in the development of varicose veins^19^. An increased IgE level is a marker of allergic disease including AR. When allergen-IgE complex binds to mast cells, the cells are activated, and release histamines and other cytokines. Elevated levels of histamines and cytokines then induce the development of varicose veins. Increased concentrations of IL-8 (interleukin-8) and MCP-1 (Monocyte chemoattractant protein-1) were noted to increase at the site of varicose veins^20^. Studies suggest that MCP-1 and IL-8 may participate in the pathogenesis of allergic rhinitis^21^. The two mechanisms could explain our findings that in pregnant women diagnosed with AR before and after pregnancy(group 3), the rate of venous complications including varicose veins increased. The increased rate of venous complications will indirectly increase the rate of TE.

Similar with asthma^9^, nasal symptoms of AR may improve (34%), worsen (15%) or remain (51%) during pregnancy^10^. AR may also occur or be noticed for the first time during pregnancy. With allergen prevention, life control and appropriate medical treatment, AR may subside or disappear after pregnancy. Decreasing the severity of allergic disease may also influence the formation of TE. In our study, compared to non-AR subjects, those with AR both before and after childbirth (group 3) had an increased rate of TE. Importantly, those whose AR was well controlled during pregnancy (group1, with AR before childbirth, but not after), did not have an increased rate of TE compared to non-AR subjects. Thus, well controlled AR during pregnancy, could decrease the rate of TE.

Allergic phenomenons activate the coagulation system^5^, enhance platelet activation^6^, and induce prothrombotic and antifibrinolytic activities through the rise of IgE^8^, and then induce the formation of TE. Histamine, an organic nitrogenous compound, is a key mediator of allergic reactions, and is a pro-coagulant too^22^. In a normal pregnancy, maternal blood histamine levels decrease during the second and third trimester^23^. Reduced histamine may decrease the symptoms of allergic disease and decrease the development of coagulation. Decreased blood histamine and well controlled allergic conditions through different methods may explain why some AR subjects had a decrease in AR symptoms after pregnancy. When AR is well controlled in patients during pregnancy, their blood histamine may decrease. Lower histamine could decrease the rate of TE. The mechanism could explain our findings that in those with AR before but no AR after childbirth (group 1, with well controlled AR), TE in pregnancy would not increase.

When AR developed or worsened during pregnancy, the rate of TE should have increased. However, the rate of TE did not increase in these subjects (group 2) in our study, compared to non-AR subjects. Hormonal changes during pregnancy can induce an increase of circulating blood volume and activity of nasal mucosa cells. It results in nasal mucosa swelling and increased secretion^10^. Although the hormonal changes can worsen the symptoms of AR, some non-AR subjects may be misdiagnosed as having AR during this period. These non-AR subjects, without allergic disease, could have been misdiagnosed as AR subjects and may have been grouped into group 2 (without AR before, with childbirth) in our study. TE induced by allergic mechanisms did not occur in these non-allergic subjects. With some non-allergic subjects being misgrouped into group 2, the rate of TE would decrease. This might explain our findings that TE was not significantly higher in group 2, compared to non-AR subjects.

There are limitations in our study. First, there is no laboratory data to evaluate the clinical condition of AR and TE. Second, there were no data in part of the AR severity (mild, moderate, severe), AR classification (intermittent, persistent, perennial or seasonal) and AR duration to show the biologic gradient on the association. There is no treatment to show the biological plausibility. Third, the temporal relationship between these two conditions could not demonstrate the temporal sequence of AR before TE due to the definitions of AR that apply the time of childbirth as a cut-off point, not the time of conception. Fourth, there is no radiological data. Radiological data is important to evaluate the profile of TE. Fifth, PE is one of the most severe diseases in TE. Evaluating the association between AR and PE in pregnant women is important. However, because patient numbers of PE were too few, it could not be calculated. Future investigation with the recruitment of more subjects, including AR classification, treatment, radiological and biological data is necessary to find the association more accurate.

## Conclusion

Through this population based, retrospective cohort study, an association between AR and TE was found in pregnant women. The association was only noted in those diagnosed with AR both before and after childbirth. An increase of venous complications in pregnancy and puerperium in these subjects could explain the increased rate of TE. This association serves to remind pregnant women with AR to exercise regularly, avoid wearing tight clothing and so on to prevent the development of venous complications such as varicose veins and TE. In those with AR before pregnancy but not after pregnancy (group 1, improved AR during pregnancy), there was no increase of TE. This result suggests that different methods, such as allergen prevention and appropriate medical treatment to improve the symptoms of AR, are important to prevent the development of TE.
